# The Effectiveness of Advanced Platelet-Rich Fibrin in comparison with Leukocyte-Platelet-Rich Fibrin on Outcome after Dentoalveolar Surgery

**DOI:** 10.1155/2021/6686857

**Published:** 2021-05-08

**Authors:** Alaa Z. Makki, Anoud M. Alsulami, Arwa S. Almatrafi, Moroj Z. Sindi, Shahinaz N. Sembawa

**Affiliations:** ^1^Oral and Maxillofacial Surgery Department, Faculty of Dental Medicine, Umm Al-qura University, Makkah 24225, Saudi Arabia; ^2^Dental and Oral Surgery, Umm Al-qura University, Makkah 24225, Saudi Arabia; ^3^Preventive Dentistry Department, Dental Public Health Division, Faculty of Dental Medicine, Umm Al-Qura University, Makkah 24225, Saudi Arabia

## Abstract

**Methods:**

The study included 60 patients according to sample size calculation, recruited from patients seeking tooth extraction at oral and maxillofacial surgery clinic at Umm Al-Qura University, Faculty of Dentistry. Patients were divided into three groups. Group Ӏ included 20 patients managed by advanced platelet-rich fibrin after extraction. Group ӀӀ included 20 patients managed by leukocyte-platelet-rich fibrin after tooth extraction. Group ӀII included 20 patients left without any addition. Each group was further subdivided into surgical and nonsurgical extraction. Afterwards, patients in each group were assessed for postextraction pain by VAS, number of analgesics, and early soft tissue healing by LWHI.

**Results:**

The study outcomes demonstrate that the use of A-PRF significantly reduces postoperative pain in the 1^st^ and 2^nd^ day. VAS pain scores on the first day were significantly higher in the control surgical extraction group and L-PRF nonsurgical extraction group. In early soft tissue healing. The Landry Wound Healing Index (LWHI) was used after 1 and 2 weeks of extraction to evaluate the extraction site. In first week, the A-PRF group and L-PRF group (nonsurgical extraction) had a better healing index when compared to control group, and A-PRF group (surgical extraction) had a best healing index when compared to L-PRF and control groups. In the second week, individuals in the A-PRF group (surgical and nonsurgical extraction) had a better healing index when compared to L-PRF and control groups.

## 1. Introduction

Extraction is the most frequent procedure done in oral and maxillofacial surgery clinics to treat periodontal disease, infections, badly decayed teeth, poorly aligned teeth, and orthodontic problems [1]. Possible postextraction complications include pain, swelling, infections, dry socket, and trismus [2].

Pain is an unpleasant sensory and emotional experience. Importantly, it is also a warning symptom of tissue damage and occurs during wound healing. It can be associated with actual or potential tissue damage [3, 4]. Pain is caused by the release of pain mediators from injured tissues, which reach their peak levels during the first postoperative day [5]. The treatment of choice for pain relief after teeth extraction is a nonsteroidal anti-inflammatory drug [6].

Healing is the repair of damaged or diseased tissue affected by a microorganism [7]. Bacteria and bacterial products can affect the processes of healing. Disturbed host-bacteria equilibrium inhibits multiple processes involved in the wound healing scheme [8].

After tooth extraction, a physiologic process is involved in soft and hard tissue healing. Postextraction healing involves several events. It starts with blood clot formation to create a matrix that guides the passage of growth factor and mesenchymal cells to activate proliferation. This is followed by differentiation and synthesis activity, with the formation of a temporary matrix after seven days that leads to temporary connective tissue with collagen formation and angiogenesis. Subsequently, there is formation of bone tissue after 14–30 days, followed by lamellar bone after 30–180 days, and bone marrow after 60–180 days [9].

There are many ways to enhance soft and hard tissue healing and maintain the volume of the tissue after extraction [10]. Many studies have indicated the advantages of using methods such as grafts, growth factors, and resorbable or nonresorbable membranes to preserve the socket [11, 12]. Autograft is associated with high degree of donor site morbidity, and allograft is associated with a risk of disease transmission. As such, clinicians have an interest in autologous materials, such as platelet-rich plasma (PRP) and platelet-rich fibrin (PRF) [13]. The main key to the healing process is the presence of adequate growth factors, which are found in platelet-rich plasma (PRP) and platelet-rich fibrin (PRF) [12]. Clinical application of PRF does not lead to immune rejection because it is obtained from the person's own venous blood. It is a second-generation product that acts as autologous growth factors. It can promote healing and is associated with early organization of bone substance. It is loaded with fibrin, platelets, white blood cells, growth factors, cytokines, and other components. PRF has all the elements of blood that release number of immune regulation related cytokine which make it promising and helpful for healing and reduce local inflammatory responses. PRF influences the proliferation, differentiation, and apoptosis of repair-related cells [1, 14].

PRF releases growth factors, such as transforming growth factor-b1 (TGF-1), platelet-derived growth factor (PDGF), and vascular endothelial growth factor (VEGF), which all have been proven to promote the wound healing and tissue regeneration [15, 16]; in addition, it releases an important coagulation glycoprotein (thrombospondin-1).

Moreover, it has a natural fibrin framework which provides protection to growth factors from proteolytic degradation. PRF releases growth factors gradually and slowly which means it is active for a relatively long period [17, 18]. Fibrin gels provide a scaffold for cell growth and a preferable environment for osteoblastic differentiation; PRF facilitates bone regeneration by space production through its strong fibrin network [19].

The use of a PRF membrane in a postextraction socket was confirmed to aid in local soft and hard tissue healing and diminishes the postoperative pain response [1, 13, 14, 20]. The effectiveness of leukocyte-platelet-rich fibrin (L-PRF) is at the same level as osseous substitutes, but without having their disadvantages, which includes remaining graft particles and high cost. L-PRF is considered an autograft of blood origin, and it does not leave residual particles in the preserved socket. The effect of L-PRF in extraction sockets is due to the release of growth factors and cytokines immersed in platelets and the fibrin mesh. They have a basic role in controlling the inflammatory response and immune system organization in the affected site. L- PRF improves cellular migration and proliferation, immune organization, and acceleration of soft and hard tissue healing. Application of L-PRF has been found to reduce postoperative pain after a third molar extraction [21]. PRF that contains a greater number of white blood cells is called advanced platelet-rich fibrin (A-PRF). Leukocytes are very important immunocytes that can direct various cell types in the process of wound healing. Leukocyte counts can be increased in the PRF matrix by reducing the centrifugal g-force. The use of A-PRF after mandibular third molar extraction significantly reduced postoperative pain and the need for analgesics [22].

PRF has gained tremendous attention in recent years due to its capacity to successfully regenerate either soft or hard tissues, enhance new blood vessel formation (angiogenesis), and support tissue formation during healing. The advantages of PRF over PRP are the lack of blood anti-coagulants, which results in a strong fibrin matrix, and considerable growth factor that may be released over a 10- to 14-day period. The combination of host cells, strong fibrin matrix, and growth factors results in faster wound healing. Three recent systematic reviews showed a beneficial effect of PRF in preventing dry sockets within the first seven days. Meta-analysis found that PRF can reduce the incidence of AO after mandibular third molar surgery in the first week and has a positive effect on reducing postoperative pain [23].

Although the ideal method for pain management after dentoalveolar surgery is still an issue of controversy, the present study investigated the hypothesis that the use of L-PRF and A-PRF could be of value as a suitable treatment for healing after dentoalveolar surgery. The aim of our study is to evaluate the effect of advanced PRF in comparison with L-PRF on soft tissue healing and pain after teeth extraction, and to provide advice to dental practitioners on the use of advanced PRF in clinic to enhance soft tissue healing and decrease pain.

## 2. Materials and Methods

This is a randomized controlled trial that explored the effect of advanced platelet-rich fibrin in comparison with leukocyte-platelet-rich fibrin on healing and postoperative pain after extraction. The protocol for the investigation was approved by an institutional review board at Umm Al-Qura University, Collage of Dentistry (UQUDENT-IRB code: 143–19). All participants were informed about the procedure and provided informed consent in English and Arabic. Data collection started in December 2019 and ended in March 2020.

### 2.1. Participants

Patients were selected from the Oral and Maxillofacial Surgery Clinic—Umm Al-qura University, Collage of Dentistry, UQUDENT, Saudi Arabia. A total of sixty subjects who required posterior tooth extraction were included in the study. Inclusion criteria consisted of patients who had surgical or nonsurgical posterior tooth extraction and can speak and communicate in Arabic or English, aged 18 or older, male or female, free of significant systemic disease, having teeth with hopeless periodontal prognosis, or with advanced carious lesions with an extraction site free of active infection.

Patients undergoing chemotherapy and radiotherapy, patients with intellectual disability, patients with medical conditions that can affect wound healing, pregnant women, and patients with distinct periapical pathology were excluded from the study. Patients who agreed to participate in the study were divided randomly into three groups.

Group Ӏ: 20 patients were managed by advanced platelet-rich fibrin after tooth extraction. Group ӀӀ: 20 patients managed by leukocyte-platelet-rich fibrin after tooth extraction, and Group ӀII: 20 patients left without any addition. Each group was further subdivided into surgical and nonsurgical extraction. Afterwards, patients in each group were assessed for postextraction pain by visual analogue scale (VAS) [22], number of analgesics, and early soft tissue healing by the Landry Wound Healing Index (LWHI) [10].

### 2.2. Study Procedure

Teeth were extracted under local anesthesia, granulation tissue was removed, and the socket was washed with normal saline 0.9 % *w*/*v* sodium chloride after extraction.

Preparation of L-PRF and A-PRF: 10 ml venous blood sample was collected from the participant and quickly transferred to glass tubes. The tubes did not contain any anticoagulant to initiate platelet activation and fibrin polymerization. The tube for the L-PRF group was centrifuged in a Medifuge™ Small Benchtop Centrifuge at 2700 rpm for 12 minutes. For the A-PRF group, the tube was centrifuged in a Medifuge™ Small Benchtop Centrifuge at 1500 rpm for 14 min.

**Figure 1:**
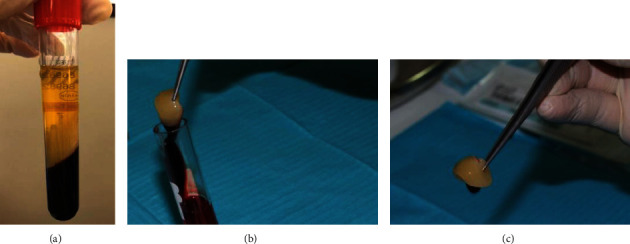
(a) Blood sample after centrifugation. (b) Extracting the PRF clot from the tube with sterile tweezer. (c) The fibrin clot is separated from the RBC fragment.

The resultant product in each tube consisted of the following three layers: topmost layer consisting of acellular plasma, PRF clot in the middle, and red blood cells at the bottom. The PRF in the middle layer was squeezed between sterile moistened sponges and the membrane was separated from the serum (Figure 1).

**Figure 2:**
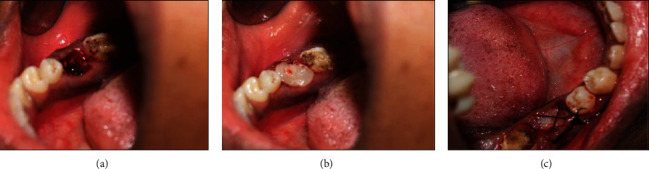
(a) Extraction socket. (b) PRF membrane filled in the socket. (c) Figure-eight suture placed.

In the test groups, L-PRF or A-PRF membranes were firmly placed in the extraction socket and then sutured with black silk 4-0 by the figure-eight technique (Figure 2).

No additional material was placed in the control group, and the socket was sutured if the extraction was surgical.

### 2.3. Evaluation Procedures

After 1 week, the suture was removed. Patients were asked to record their pain level by the visual analogue scale (VAS) at the first and second day after extraction, and the number of analgesics taken for the first day after tooth extraction.

The Landry Wound Healing Index (LWHI) was used after 1 and 2 weeks to evaluate the extraction site based on tissue color, response to touch, the marginality of the incision line, and extent of the area. The index ranges between 1 = very poor and 5 = excellent.

  1 = Very poor: ≥50% of the gingiva is red, touch causes bleeding, granulation tissue is present, the incision margin is not epithelialized, with loss of epithelium beyond the incision margin, and suppuration is present.
  2 = Poor: ≥50% of the gingiva is red, touch causes bleeding, granulation tissue is present, the incision margin is not epithelialized, and connective tissue is exposed.
  3 = Good: ≥25 to <50% of the gingiva is red, there is no bleeding on palpation, there is no granulation tissue, and no connective tissue is exposed at the incision margin.
  4 = Very good: <25% of the gingiva is red, there is no bleeding on palpation, there is no granulation tissue, and no connective tissue is exposed at the incision margin.
  5 = Excellent: All tissue is pink, there is no bleeding on palpation, there is no granulation tissue, and no connective tissue is exposed at the incision margin.

The examiners were trained by an experienced surgeon. Inter-examiner consistency and intra-examiner consistency were measured using Kappa test to ensure the validity and reliability of the data.

### 2.4. Data Statistical Analysis

Data was statistically analyzed using the Statistical Package for Social Science (SPSS v.21). *P* < 0.05 was considered significant. Descriptive statistics were used. Chi square test was used for comparisons between groups. The sample size was determined according to sample size calculation where the margin of error was 5%, confidence level 95%.

## 3. Results

### 3.1. Participants

The study included 60 patients seeking tooth extraction recruited from the Oral and Maxillofacial Surgery Clinic at the Faculty of Dentistry, Umm Al-Qura University. Patient ages ranged from 18 to 60 years. Group I included 9 male patients (4 surgical extractions, 5 nonsurgical extractions) and 11 female patients (5 surgical extractions, 6 nonsurgical extractions), and Group II included 10 males patients (4 surgical extractions, 6 nonsurgical extractions) and 10 female patients (4 surgical extractions, 6 nonsurgical extractions), while Group III included 10 male patients (4 surgical extractions, 6 nonsurgical) and 10 female patients (4 surgical, 6 nonsurgical extractions).

### 3.2. Results of Visual Analogue Scale Pain Scores

In this study, we assessed the pain on the first and second days of tooth extraction by using the visual analogue scale. VAS pain scores on the first day were significantly higher in the control surgical extraction group and L-PRF nonsurgical extraction group (*P*=0.005), while the A-PRF group showed mild pain scores in the surgical extraction group, and no pain scores in nonsurgical extraction group (Table 1).

On the second day, there were significantly higher scores in the control group than the other two groups (*P* ≤ 0.001), the L-PRF group showed mild pain scores in the nonsurgical extraction group, and the PRF group showed no pain scores in the nonsurgical extraction group (Table 2).

### 3.3. Results for Number of Analgesics Taken by the Patients

**Figure 3:**
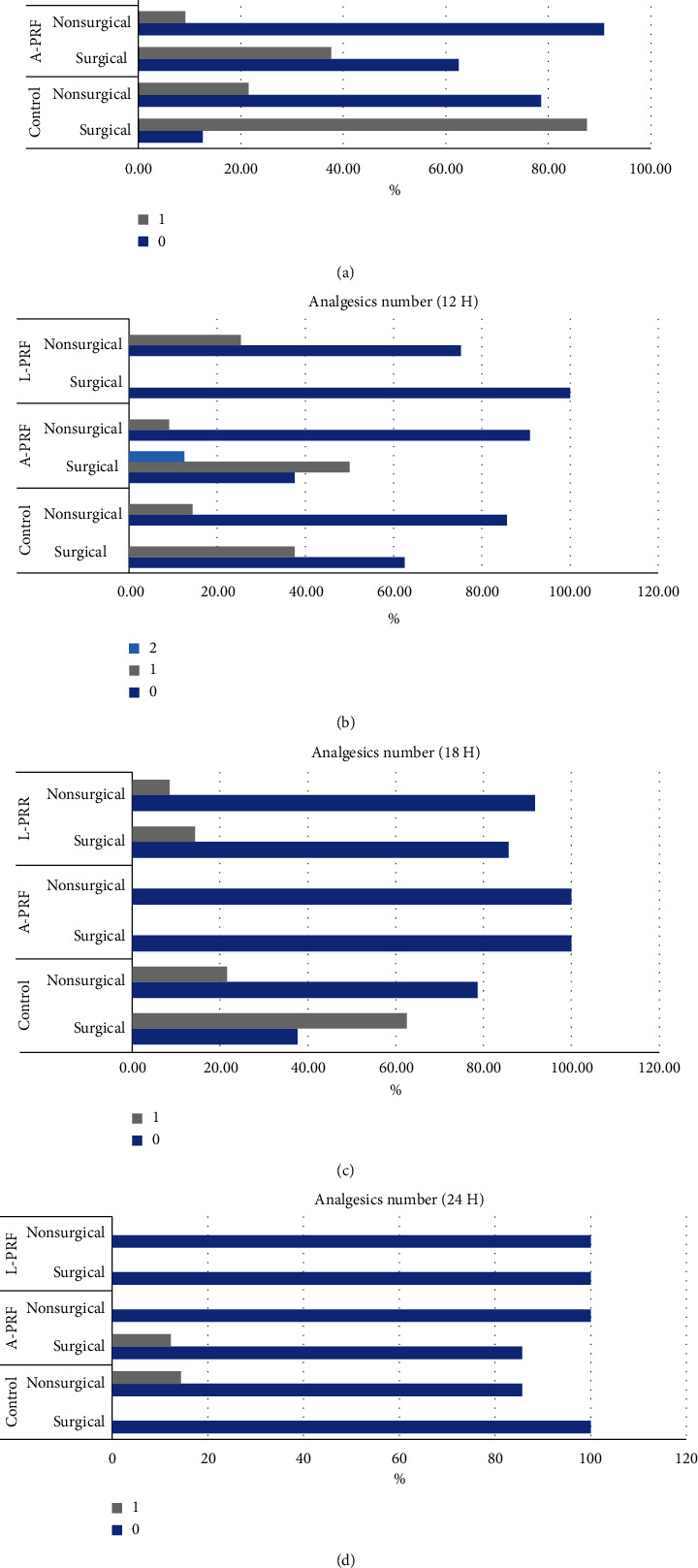
(a) Analgesics number (6 H). A-PRF = advanced platelet-rich fibrin, L-PRF = leukocyte-platelet-rich fibrin. 1 (grey), 0 (blue) represent number of analgesics. (b) Analgesics number (12 H). A-PRF = advanced platelet-rich fibrin, L-PRF = leukocyte-platelet-rich fibrin. 1 (grey), 0 (blue), 2 (light blue) represent number of analgesics. (c) A-PRF = advanced platelet-rich fibrin, L-PRF = leukocyte-platelet-rich fibrin. Analgesics number (18 H). 1 (grey), 0 (blue) represent number of analgesics. (d). Analgesics number (24 H). A-PRF = advanced platelet-rich fibrin, L-PRF = leukocyte-platelet-rich fibrin. 1 (grey), 0 (blue), 2 (light blue) represent number of analgesics.

Patients were asked about the number of analgesics that were taken in the first (6, 12, 18, 24) hours after tooth extraction. In the first six hours, the number of analgesics taken by the patients was significantly higher in the control surgical extraction group (*P*=0.01) (Figure 3(a)).

There was no significant difference between groups in terms of the number of analgesics in the first 12 hours (*P*=0.1) (Figure 3(b)). In 18 hours, the number of analgesics taken was significantly higher in the control surgical extraction group (*P*=0.005) (Figure 3(c)), while at 24 hours there was no significant difference between the groups (*P*=0.3) (Figure 3(d)).

### 3.4. Results of Soft Tissue Healing

The Landry Wound Healing Index (LWHI) was used after 1 and 2 weeks of extraction to evaluate the extraction site based on tissue color, response to touch, the marginality of the incision line, and extent of the area. In the first week, patients in the A-PRF (surgical group) had the best outcome; A-PRF and L-PRF group (nonsurgical extraction) had a better healing index when compared to control groups (*P* ≤ 0.001) (Table 3).

In the second week, individuals in A-PRF group (surgical and nonsurgical extraction) had a better healing index when compared to L-PRF and control group (*P* ≤ 0.001) (Table 4).

## 4. Discussion

The primary aim of this study was to compare the postoperative effects of L-PRF and A-PRF on pain, total number of painkillers (analgesics) taken, and healing of soft tissue after extraction. Our findings suggest that the A-PRF group showed a significant difference in pain score on the first and second day, number of analgesics in the first 6 and 18 hours, and soft tissue healing in the first and second week after tooth extraction compared to other groups.

The study outcomes demonstrate that the use of A-PRF significantly reduced postoperative pain and the patients need to take analgesics and enhanced early soft tissue healing of A-PRF group compared to L-PRF group.

Caymaz and Uyanik [22] who conducted a study to investigate and compare the postoperative effects of leukocyte-platelet-rich fibrin (L-PRF) and advanced platelet-rich fibrin (A-PRF) in terms of pain, swelling, and trismus after mandibular third molar surgery found that the postoperative pain score and number of painkillers were significantly reduced in A-PRF when compared with L-PRF [22]. These findings are in agreement with the finding of the current study.

Several studies used the same method used in the current study [24, 25], while other studies used different methods such as different PRF preparation [26]. Fujioka-Kobayashi et al. prepared A-PRF at 1300 rpm for 14 minutes, while Caymaz and Uyanik prepared L-PRF at 3000 rpm for 10 minutes [22].

Recently, many studies found that modifications to centrifugation speed and time, by reducing the centrifugal g-force, is favorable, because it increases leukocyte counts, and platelet and growth factor release from PRF clots [26, 27]. Reducing the force during centrifugation results in a higher cell number. A- PRF might influence soft tissue and bone regeneration, through the presence of monocytes/macrophages and their growth factors. In the current study, the A-PRF group was centrifuged at 1500 rpm for 14 minutes, while the L-PRF group was centrifuged at 2700 rpm for 12 minutes.

In the literature, there are three clinical studies that compared between L-PRF and A-PRF.

The results of one of these studies showed that the postoperative pain score and number of painkillers were significantly reduced in A-PRF when compared with L-PRF [22].

Other studies compared between A-PRF and L-PRF regarding the release of growth factors [25, 26] They found that A-PRF releases a significantly higher amount of growth factors compared with L- PRF [25, 26].

Many studies have proven that the concentration of platelets and leukocytes plays a central role in provoking the healing and regenerative process of the tissue by releasing growth factors and cytokines [28]. The second generation (L-PRF and A-PRF) differs from the first generation (PRP) in leukocyte content and amount of growth factor [24].

In the A-PRF, the number of leucocytes includes more neutrophils, which can help in differentiation of monocyte/macrophage [27]. Also, A-PRF has higher amounts of platelets and releases a significantly higher quantity of growth factors as TGF-*β*, PDGF, VEGF, and chemotactic molecules compared to the original PRF [25]. This is due to the low speed of centrifugation which can clinically translate into an increased concentration of growth factors and neoangiogenic potential. As such, A- PRF is considered as an excellent candidate for tissue regeneration [29].

This study had several strengths. In particular, this was a randomized controlled trial, which is considered the gold standard in medical research. The sample size was a relatively large sample size compared to previous studies [1, 22]. Our study is also the first study in Saudi Arabia to compare the effects of A-PRF and L-PRF in postoperative pain and at an early stage of soft tissue healing after an extraction. This study had some limitations. The indices that were used are subjective. Some of the age groups were difficult to find in good systemic health. The study was conducted in Umm Al-Qura University, Collage of Dentistry (UQUDENT) clinics, and considered the first one. Extractions were not done by the same surgeon, which affects the homogeneity of each group. We recommend for researchers who are interested in this topic to have the extractions done by the same surgeon to ensure homogeneity. Further clinical trials are needed to measure bone healing.

## 5. Conclusion

In conclusion, this study showed that the use of a simple, cost-effective A-PRF significantly reduced postoperative pain and the need for analgesics and enhanced soft tissue healing at extraction sockets. The effects of these biomaterials on soft and hard tissues should be further evaluated in other clinical trials for a longer period and in a larger sample.

## Figures and Tables

**Table 1:** The visual analogue scale pain scores of each group on the first day after tooth extraction.

Pain 1^st^ day	Control		A-PRF		L-PRF	
	Surgical (%)	Nonsurgical (%)	Surgical (%)	Nonsurgical (%)	Surgical (%)	Nonsurgical (%)
No pain	0.0	0.0	0.0	27.3	0.0	0.0
Mild	12.5	14.3	50.0	45.5	42.9	16.7
Moderate	37.5	64.3	25.0	27.3	28.6	66.7
Severe	12.5	14.3	25.0	0.0	28.6	16.7
Worst	37.5	7.1	0.0	0.0	0.0	0.0
*P*	40.065 (0.005)					

A-PRF = advanced platelet-rich fibrin, L-PRF = leukocyte-platelet-rich fibrin, and *P* = *P* value, calculated by chi square test.

**Table 2:** The visual analogue scale pain scores of each group on the second day after tooth extraction.

	Control		A-PRF		L-PRF	
Pain 2^nd^ day	Surgical (%)	Nonsurgical (%)	Surgical (%)	Nonsurgical (%)	Surgical (%)	Nonsurgical (%)
No pain	0.0	0.0	25.0	63.6	14.3	8.3
Mild	12.5	28.6	50.0	36.4	42.9	66.7
Moderate	62.5	64.3	25.0	0.0	42.9	25.0
Severe	25.0	7.1	0.0	0.0	0.0	0.0
Worst	0.0	0.0	0.0	0.0	0.0	0.0
*P*	39.258 (0.001)					

A-PRF = advanced platelet-rich fibrin, L-PRF = leukocyte-platelet-rich fibrin, and *P* = *P* value, calculated by chi square test.

**Table 3:** Landry Wound Healing Indexes of each group on the first week after tooth extraction.

	Control		A-PRF		L-PRF	
Soft tissue healing 1^st^ week	Surgical (%)	Nonsurgical (%)	Surgical (%)	Nonsurgical (%)	Surgical (%)	Nonsurgical (%)
Very poor	0.0	7.1	0.0	0.0	0.0	0.0
Poor	75.0	57.1	0.0	0.0	14.3	8.3
Good	25.0	28.6	0.0	27.3	57.1	50.0
Very good	0	7.1	100	63.6	28.6	25.0
Excellent	0	0	0.0	9.1	0.0	16.7
*P*	51.664 (≤0.001)					

A-PRF = advanced platelet-rich fibrin, L-PRF = leukocyte-platelet-rich fibrin, and *P* = *P* value, calculated by chi square test.

**Table 4:** Landry Wound Healing Indexes of each group on the first week after tooth extraction.

	Control		A-PRF		L-PRF	
Soft tissue healing 2^nd^ week	Surgical (%)	Nonsurgical (%)	Surgical (%)	Nonsurgical (%)	Surgical (%)	Nonsurgical (%)
Very poor	0.0	0.0	0.0	0.0	0.0	0.0
Poor	25.0	35.7	0.0	0.0	0.0	0.0
Good	75.0	42.9	0.0	0.0	28.6	16.7
Very good	0.0	21.4	25.0	36.4	42.9	28.6
Excellent	0.0	0.0	75.0	63.6	28.6	41.7
*P*	45.389 (≤0.001)					

A-PRF = advanced platelet-rich fibrin, L-PRF = leukocyte-platelet-rich fibrin, and *P* = *P* value, calculated by chi square test.

## Data Availability

The data used to support the findings of this study are available from the corresponding author upon request.
